# A novel recombinant *Theileria annulata* surface protein as an antigen in indirect enzyme-linked immunosorbent assay for the serological diagnosis of tropical theileriosis

**DOI:** 10.14202/vetworld.2024.1936-1942

**Published:** 2024-08-27

**Authors:** Anara Ryskeldina, Aleksandr Korobeinikov, Nailya Tursunbay, Maxat Berdikulov, Alexandr Shevtsov, Christian Bauer, Yersyn Mukhanbetkaliyev, Marat Kuibagarov

**Affiliations:** 1 National Center for Biotechnology, 01000, Astana, Kazakhstan; 2 S. Seifullin Kazakh Agro Technical Research University, 010011, Astana, Kazakhstan; 3 National Veterinary Reference Center, 010000, Astana, Kazakhstan; 4 Institute of Parasitology, Justus Liebig University Giessen, 35392, Giessen, Germany

**Keywords:** cattle, indirect enzyme-linked immunosorbent assay, Kazakhstan, recombinant *T. annulata* surface protein, *Theileria annulata*

## Abstract

**Background and Aim:**

*Theileria annulata* infection in cattle causes major economic losses in livestock production in many Central Asian countries, including the southern region of Kazakhstan. This study aimed to obtain a recombinant *T. annulata* surface protein (TaSP) and to investigate its possible use as an antigen in an indirect enzyme-linked immunosorbent assay (ELISA) for the serological diagnosis of bovine theileriosis.

**Materials and Methods:**

Recombinant TaSP was obtained by cloning a polymorphic region of the TaSP gene, expressing it in *Escherichia coli* strain BL21, and purifying it by metal chelating chromatography. An indirect ELISA using recombinant TaSP as an antigen was developed and evaluated for the detection of *T. annulata*-specific antibodies in plasma samples from 69 cows polymerase chain reaction (PCR)-positive or PCR-negative for *T. annulata* and/or *Theileria orientalis* from southern Kazakhstan.

**Results:**

The obtained recombinant protein had a molecular weight of 32 kDa, and mass spectrometry analysis of the purified protein identified it as a fragment of the surface protein of *T. annulata*. Initial testing of 69 field plasma samples from cattle showed that the results of indirect ELISA using TaSP as an antigen agreed substantially with those of *T. annulata* PCR (κ: 0.78). The relative sensitivity and specificity of indirect ELISA were 88.7% and 100%, respectively, using PCR as a reference. There was no evidence of cross-reaction with *T. orientalis*.

**Conclusion:**

Initial results using recombinant TaSP as an antigen in indirect ELISA are promising and support the widespread use of this assay for routine diagnosis and *T. annulata* seroprevalence studies in cattle in Kazakhstan and possibly neighboring countries.

## Introduction

*Theileria* species (Piroplasmida) are among the most economically important hemoparasites of cattle and other bovines and are transmitted by hard ticks. In the mammalian host, cells first multiply in lymphocytic cells and then in erythrocytes [1]. *Theileria parva*, which is transmitted by *Rhipicephalus* ticks and found in sub-Saharan Africa, is the most pathogenic species, causing “East Coast fever” with up to 100% mortality in susceptible animals [2]. *Theileria annulata* is transmitted by ticks of the genus *Hyalomma* and is geographically distributed throughout the Mediterranean, Middle East, and Asia. Infection with this species is usually less severe; it causes a chronic leukoproliferative disease called “tropical theileriosis,” which is characterized by swollen lymph nodes, fever, and wasting. However, it can also be fatal [1, 3]. *T. orientalis*, which is transmitted by *Haemaphysalis* ticks, is a species with the widest geographical distribution, including Eurasia and Australia. It is usually low pathogenic, but certain genotypes can cause a significant disease called “oriental theileriosis” [4].

In Central Asia, infections by both *T. annulata* and *T. orientalis* are highly prevalent in Kyrgyzstan [5–7] and the southern regions of Kazakhstan [8]. In Kazakhstan, for example, the high prevalence of *T. annulata* infection has hindered the intensification of cattle farming, especially as more productive cattle breeds are becoming more susceptible to infection. There is also a possibility that this pathogen could spread to new regions through the movement of cattle, as vector ticks can occur in large parts of the country, with the exception of northern regions [9–11]. Testing cattle before moving to other regions could limit or prevent the spread of infection using rapid and reliable methods.

In Kazakhstan, routine diagnosis of theileriosis is based on microscopic detection of *Theileria* stages in blood. Although this method is relatively simple and allows etiological diagnosis in acute disease, it has low sensitivity [12]. There have been a number of successful research studies using polymerase chain reaction (PCR) to detect infection with *T. annulata* and other piroplasmid parasites at very low levels of infection [5, 7, 8, 12–15]. However, the use of PCR for routine diagnosis is still limited in many countries because of the need for well-equipped laboratories and the relatively high cost. In these countries, tests that detect pathogen-specific antibodies in host plasma or serum samples are more suitable for routine use because they are relatively inexpensive, easy to perform, and reproducible. One serological method for detecting *T. annulata* infection is the indirect fluorescent antibody test (IFAT) using the schizont or merozoite antigens of the parasite. Although IFAT is sensitive and usually easy to perform, it is not suitable for diagnosing *T. annulata* in regions where different *Theileria* spp. occur because of frequent cross-reactivity with other species [13, 16]. The first developed enzyme immunoassays (enzyme-linked immunosorbent assay [ELISAs]) were also based on the use of *Theileria* schizont or merozoite antigens [17, 18]. Their use has also been limited by frequent cross-reactivity between different *Theileria* spp., difficulties in standardizing the antigens, and the need to produce antigens from experimentally infected animals. These problems have been overcome by the generation of recombinant *Theileria* antigens.

*T. annulata* surface protein (TaSP) is a popular target for the production of recombinant antigens. TaSP is expressed in the sporozoite and schizont stages of this pathogen. It is highly immunogenic, and epitopes are present in the polymorphic region of the TaSP gene sequence and are distributed in different genotypes of *T. annulata* [19–21]. Despite the successful use of recombinant antigens in the serological diagnosis of bovine theileriosis, commercial test system offerings are lacking. The first commercial ELISA (Svanovir *Theileria annulata*-Ab, Boehringer Ingelheim Svanova, Uppsala, Sweden) based on recombinant TaSP was launched in 2015, but no information is currently available on the manufacturer’s website [22]. A few similar test systems have been offered by other companies (Bovine *Theileria annulata* ELISA Kit’, Gentaur, USA, ‘Cow *Theileria* Antibody ELISA Kit’, Abbexa, USA) [23, 24].The polymorphic TaSP region is variable [21]; thus, a recombinant antigen designed for one country or continent may not be suitable for another country or continent. Therefore, this study aimed to identify a recombinant TaSP as an antigen specifically tailored for tropical theileriosis diagnostics in Kazakhstan and to develop an ELISA based on this antigen.

## Material and Methods

### Ethical approval

No ethical approval was required for this study because the blood samples were collected during official surveillance. A trained person collected the blood samples using standard collection techniques without harming animals.

### Study period and location

Whole blood samples were collected during September and October-2022 from Turkistan province of Kazakhstan. The samples were processed at Applied Genetics Laboratory of National Center for Biotechnology.

### Sampling

On the respective sampling day, all village cattle were driven into a paddock before grazing. The animals appeared healthy on inspection, but no specific clinical examination was performed. Samples were collected from cattle aged 3 years or older. Whole blood samples (4 mL) were collected from 69 cows from 4 settlements in the Turkistan province of Kazakhstan. Blood was collected in vacuum tubes with ethylenediamine tetra-acetic acid (EDTA) and transported at a temperature of 4°C to the laboratory within 48 h.

### Preparation of recombinant TaSP

The polymorphic region of TaSP, located on the outer surface of the cell membrane of *T. annulata* macroschizonts [21], was selected for antigen preparation and initial screening of bovine plasma samples through indirect ELISA.

The nucleotide sequence encoding this protein fragment was amplified from *T. annulata* genomic DNA and cloned into the pET19b vector using the ligase-free method (site-directed, ligase-independent mutagenesis [SLIM]) [25]. Primers for PCR were designed using PrimerSelect (DNASTAR), BioEdit, and National Center for Biotechnology Information (NCBI) PrimerBlast (http://primer3.sourceforge.net/primer3_manual.htm. BioEdit is a biological sequence alignment editor, Version: 7.7.1 [×86]). The following key parameters were considered while selecting primers: Identical annealing temperatures for forward and reverse primers, primer length, and low probability of secondary structure formation. The novel primers used are listed in Table-1.

**Table-1:** Primer pairs used for constructing pET-19b-TaSP.

Target	Primer name	Primer sequences (5’–3’)
*T. annulata*, TaSP	TASP-Fw2	GATCGACAACTTAATCCTATCGATTTTG
	TASP-Rv2	TTTTCCGTCAGAATCATCATCATGG
*T. annulata*, TaSP	TASP-pET19b-Fw1	ACGACGACAAGCATGATCGACAACTTAATCCTATCGATTTTG
	TASP-pET19b-Rv1	GATCACCTCGAGTCATTTTCCGTCAGAATCATCATCATGG
pET19b	pET19b -Fw2	CGGCTGCTAACAAAGCCCG
	pET19b -Rv2	CGTCGATATGGCCGCTGCTG
pET19b	pET19b -Rv2	CGTCGATATGGCCGCTGCTG
	pET19b -Fw1	TGACTCGAGGTGATCCGGCTGCTAACAAAGCCCG

*T. annulata=Theileria annulata*

A single PCR was performed for each modification and contained the following components: 4 μL of 5× Phusion High-Fidelity (HF) buffer, 2 mM dNTP, 10 pmol of each primer, 100 pg of plasmid template pET19b, 0.5 U Phusion® HF DNA Polymerase (Thermo Scientific, Baltics UAB, cat. F-530L, Waltham, Massachusetts, USA), and molecular biology grade water to a final volume of 20 μL. PCR was performed at 98°C for 1 min, 25 cycles of 98°C for 10 s, 60°C for 40 s, and 68°C for 3 min, with a final extension step at 68°C for 10 min. All four PCR mixtures were then diluted and mixed with 5 U DpnI enzyme (New England Biolab, cat. R0176S, Frankfurt am Main, Germany). This mixture was incubated at 37°C for 60 min. DpnI digestion was stopped by denaturation at 95°C for 3 min. Hybridization was performed by two cycles of 65°C for 5 min and 30°C for 15 min.

The resulting construct was transformed into XL-Blue cells (Agilent Technologies, cat. 200249, Novogene, Cambridge, UK) using the Hanahan method, followed by colony selection and plasmid expansion [26]. The resulting plasmid was used to transform electrocompetent BL21 (DE3) cells (Agilent Technologies, cat. 200133, Novogene) through electroporation using the GenePulser Xcell (BioRad, cat. 1652660, Hercules USA.) under the following conditions: 100 ng of plasmid per 50 μL of cells, voltage 2.5 kV; electrical capacitance 25 μF, resistance 200 Ohm. The transfer time was 5.2 ms. Transformed cells were incubated in 950 μL of Super Optimal broth with Catabolite repression medium (SOC medium) at 37°C for 1 h with vigorous shaking. Then 50 μL of cells were plated on Luria-Bertani (LB) agar with antibiotic as a selection factor and grown at 37°C for 16 h. Single colonies of transformant were grown in LB broth containing antibiotics. In the middle of the logarithmic phase of bacterial mass growth (OD600=0.6), an inducer, isopropyl-β-D-1-galactopyranoside (IPTG) was added at a final concentration of 1 mM and incubated for 4 h. Cells were harvested through centrifugation at 4°C and 5000× *g* for 10 min.

Cell lysis was performed using a Sonic Ruptor 4000 ultrasonic disruptor (Omni International, cat. 230-4103-OMN, Georgia, USA) at a frequency of 24 kHz in pulsed mode (10 pulses, 10 s/pulse) on ice in lysis buffer (50 mM Na_2_HPO_4_, 300 mM NaCl, 10 mM imidazole, pH 8.0). Protein purification was performed by metal chelating chromatography using a HiTrap Chelating HP 1 mL column (GE Healthcare, USA) and an AKTA purifier 10FPLC system (GE Healthcare, USA). Column equilibration and lysate loading were performed according to the manufacturer’s protocol. To determine the concentration of the elution solution, a stepwise gradient of imidazole was used with initial buffer A (50 mM Na_2_HPO_4_, 300 mM NaCl, 20 mM imidazole, pH 8.0) and final buffer B (50 mM Na_2_HPO_4_, 300 mM NaCl, 250 mM imidazole, pH 8.0). Protein concentrations in the lysates and fractions were determined using the Bradford method with bovine serum albumin as the standard [27]. Electrophoretic separation of the recombinant protein was performed using the Laemmli method [28] on a polyacrylamide gel (15%) under denaturing conditions.

*Escherichia coli* BL21 AI™ (Invitrogen, Waltham, Massachusetts, USA) cells were transformed with pET-19b-TASP and grown in an LB/ampicillin medium. IPTG was added to the biomass at a concentration of 1 mM. For expression analysis, the BL21 (DE3)/pET-19b_TASP expression strain culture was grown to OD600=0.6. As a negative control, an *E. coli* culture of the same strain without the plasmid was grown without induction. After 4 h of induction, 1 mL of each culture was harvested. The cell biomass was pelleted, disrupted by sonication, and clarified by centrifugation. The supernatants and pellets were mixed separately with Laemmli loading buffer and analyzed by sodium dodecyl-sulfate polyacrylamide gel electrophoresis.

For the analysis of the recombinant protein, we used an Impact II chromatography-mass spectrometer (Bruker, Mundelein, Illinois, USA) with a Dionex Ultimate 3000 RSLCnano (Thermo Scientific, With nano ProFlow flow meter: 900 bar [13,050 psi], USA) high-performance liquid chromatography system with separation on an Acclaim Pep-Map RSLC (Acclaim Pep-Map RSLC 164946, Trap Column, Particle Size 3 μm, Thermo Scientific™, Waltham, Massachusetts, USA) column using an acetonitrile/water solvent gradient and 0.1% formic acid. The obtained data were analyzed using Mascot software (Matrix Science with SwissProt and NCBI databases, National Library of Medicine, NIH, Washington, DC, USA).

### Development of indirect ELISA using recombinant TaSP

To avoid possible non-specific binding of antibodies, plasma samples were incubated with an adsorption solution prepared according to Jaramillo Ortiz *et al*. [29] with slight modifications (We used the adsorption solution as a buffer to directly dilute samples into the antigen wells, which reduced the reaction time). Briefly, 500 mL of an overnight culture of *E. coli* strain BL21 AI™ (Novogene, Cambridge, UK) was centrifuged at 6640 rcf for 10 min at 4°C. The sediment was resuspended in 40 mL lysis buffer (100 mM Tris HCl, pH: 7.5; 500 mM NaCl; 20% glycerol; 1% Triton X-100; 20 mM imidazole pH: 7.4; 1 mg/mL lysozyme; 0.5 mM phenylmethylsulphonyl fluoride) for 2 h at 4°C with gentle stirring. The suspension was treated with ultrasound in three cycles (1 min/cycle) and centrifuged at 6640× *g* for 30 min at 4°C. The supernatant (lysate) was collected, the protein concentration was determined, and the solution was frozen at –80°C until use. The adsorption solution was prepared by diluting the lysate to a final concentration of 1000 μg/mL in 1X tris-buffered saline and 0.1% Tween^®^ 20 detergent (Tris-buffered saline [TBS]-T, Bio-Rad, 1705017, Hercules, USA). In a preliminary experiment, the buffer containing the lysate was tested against a buffer containing TBS-T alone for the dilution of plasma samples using samples from 10 and 5 *T. annulata* DNA-positive and DNA-negative cattle. The use of the buffer with lysate prevented the generation of false-positive results compared with the buffer without lysate (Supplementary data). Therefore, in further work, a buffer containing lysate was used to dilute blood plasma samples to avoid possible non-specific binding of antibodies.

Wells of 96-well plates for immunological reactions (Aptaca, 96-well plates, 5096/P. Canelli, Italy) were coated with recombinant TaSP as antigen in carbonate-bicarbonate buffer (pH 9.5) at a concentration of 2 μg/mL and incubated overnight at 4°C. To remove unbound antigens, the plates were washed three times with TBS-T. The blocking solution used was 5% nonfat dry milk (Oxoid, LP 0031, Waltham, Massachusetts, USA) dissolved in TBS-T. A further 90 μL of the adsorption solution and 10 μL of each plasma sample (final dilution 1/10) were added, and the samples were incubated for 2 h at 37°C on a shaker. After incubation, the plates were washed three times with TBS-T to remove non-specifically bound antibodies. Horseradish peroxidase-labeled anti-species antibodies (anti-bovine IgG HRP, Sigma, A5295, Saint Louis, USA) were then added to each well and incubated at 37°C for 1 h. The washing procedure was repeated to remove unbound reaction products and 100 μL of tetramethylbenzidine enzyme substrate solution (TMB, Immunotek, Russia) was added to each well. The plates were incubated at room temperature (25°C) for 10 min. A positive reaction is indicated by the substrate solution turning blue. The reaction was stopped by adding 100 μL of 3M HCl solution to each well. Optical densities (OD) were measured using a Stat Fax 4300 (ChroMate, Awareness Technology, Palm City, Florida, USA) spectrophotometer at 450 nm.

Finally, the final cutoff value was determined using 20 plasma samples from cattle that were negative for *T. annulata, T. orientalis*, and *Babesia* spp. in a known piroplasmid-free region of Kazakhstan. To minimize false positives, the cutoff value was calculated as the mean OD plus two standard deviations of the samples. This resulted in an optimized cutoff value of 0.164 for discriminating between positive and negative results.

### Use of indirect ELISA on field samples

Total DNA extraction from whole blood and PCR were performed on all samples using piroplasmid species- or genus-specific primers as described by Kuibagarov *et al*. [8]. Of these cattle, 53 were identified as *T. annulata* DNA-positive and 30 as *T. orientalis* DNA-positive for *T. orientalis*, but all were DNA-negative for *Babesia* spp. (Supplementary data). Indirect ELISA using the novel recombinant TaSP as antigen was then evaluated as the first application for the detection of *T. annulata*-specific antibodies in plasma samples from these 69 cattle using a final cutoff of 0.164. The relative sensitivity and specificity of the ELISA, including their 95% confidence intervals (95% CI), were determined using the results of *T. annulata* PCR as the reference test; the *kappa* statistic was calculated to assess the agreement between the results of the ELISA and the PCR [30].

## Results

**Figure-1:**
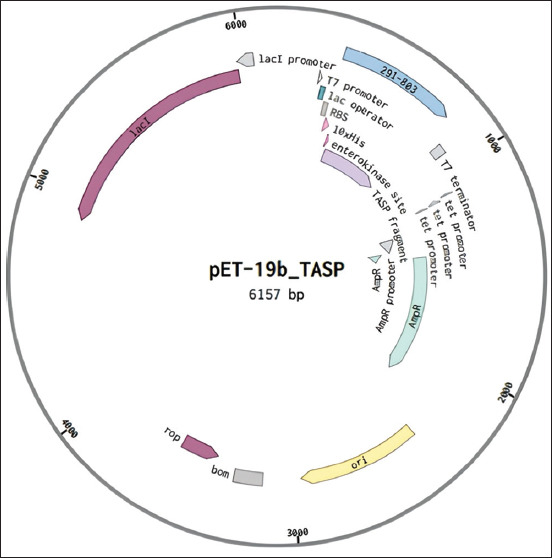
Plasmid map of the bacterial expression of the *Theileria annulata* surface protein.

**Figure-2:**
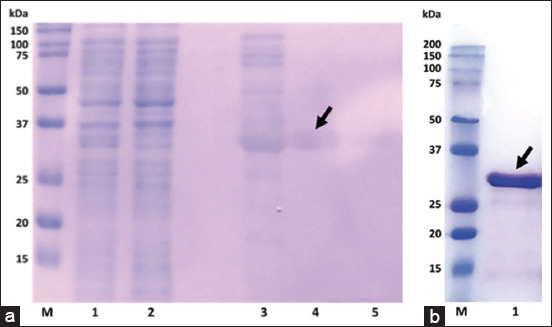
Sodium dodecyl-sulfate page results of the recombinant *Theileria annulata* surface protein. (a) Lane M shows the DNA size marker (Precision Plus Protein™ Dual Color Standards; Bio-Rad, USA, cat. 1610394); lanes 1 and 2 show the lysate and flow-through, respectively; lanes 3–5 show protein fractions #1, #2, and #3, respectively. (b) Lane M shows the DNA size marker (Precision Plus Protein™ Dual Color Standards; Bio-Rad, USA, cat. 1610394); lane 1 shows the protein after large-scale isolation. The arrows indicate the target band.

The TaSP gene fragment was successfully cloned into the pET-19b expression vector, resulting in the pET-19b_TASP (6157 bp) vector (Figure-1). After optimizing the culture conditions, the maximum protein expression was observed at 37°C with 1 mM IPTG for 6 h. As a result of transformation and expression, the production of the target antigen with a molecular weight of 32 kDa was observed in the bacterial lysate, with the protein remaining in the soluble fraction. Purification by metal chelate chromatography on nickel-Sepharose gave a good yield of pure recombinant TaSP with a molecular weight of 32 kDa in 3 fractions (Figures-2a and b). The optimal imidazole concentration during elution was determined to be 250 mM.

Mass spectrometry data of the recombinant protein showed the maximum score for the *T. annulata* macroschizont stage TaSP protein.

Using 69 *T. annulata* DNA-positive and -negative bovine field blood samples, the indirect ELISA results substantially agreed with those of *T. annulata* PCR (kappa value: 0.78). The relative sensitivity and specificity of ELISA were 88.7% and 100%, respectively, using PCR as a reference. The positive and negative predictive values were 100% and the negative predictive value was 72.7%. The ELISA accuracy was 91.3% (Table-2). Plasma samples from two *T. orientalis* PCR-positive but *T. annulata* PCR-negative cattle were negative by ELISA, indicating no cross-reaction with *T. orientalis* (Supplementary Table-S2).

**Table-2:** Comparison of the results of indirect ELISA using a novel recombinant *T. annulata* surface protein as antigen with those of *T. annulata*-specific PCR as the reference test using 69 bovine blood samples from the Turkistan province of Kazakhstan.

Indirect ELISA	PCR	

	Positive	Negative
Positive	47	0
Negative	6	16
Relative sensitivity (%) of ELISA: 88.7 (76.9–95.3)^a^		
Relative specificity (%) of ELISA: 100 (79.4–100)		
Positive predictive value (%) of ELISA: 100 (92.4–100)		
Negative predictive value (%) of ELISA: 72.7 (49.8–89.3)		
Accuracy (%) of ELISA: 91.3 (82.0–96.7)		
k: 0.78 (0.62–0.94)		

a 95% confidence interval. PCR=Polymerase chain reaction, ELISA=Enzyme-linked immunosorbent assay, *T. annulata*=*Theileria annulata*

## Discussion

The aim of the present study was to develop an optimal ELISA protocol using a novel recombinant TaSP antigen for the serological diagnosis of *T. annulata* infection in cattle. We obtained a recombinant protein fragment of TaSP, as previously proposed by Schnittger *et al*. [19]. We used an indigenous *T. annulata* strain circulating in Kazakhstan as the sequence used. Our cloned natural sequence differed by 46 amino acid substitutions, including 36 deletions, from the sequence proposed by Schnittger *et al*. [19] (accession number AJ316250). The closest sequence was published by Pain *et al*. [31] (accession number XM947650), which differs by seven amino acid substitutions. This result confirms the high polymorphism degree of the TaSP fragment reported in the literature. Our results also showed that the strategy used to obtain the recombinant protein was successful: mass spectrometry analysis revealed TaSP in the *T. annulata* macroschizont stage with high identity. The use of this protein as an antigen in ELISA permitted the detection of *T. annulata*-specific antibodies in plasma samples from infected cattle. The optimized ELISA protocol included the previously proposed principle of pre-adsorption of blood plasma samples with lysate from the expressed *E. coli* strain [29, 32]. However, unlike previous protocols, we did not pre-incubate the plasma samples. Instead, we used the adsorption solution as a buffer to directly dilute the samples into the antigen wells, which shortened the reaction time. The use of this adsorption strategy resulted in a reduction in the optical density of the negative plasma samples (Supplementary Table-S1).

As previously reported by Al-Hosary *et al*. [22] and Renneker *et al*. [33], TaSP-based ELISAs are prone to sensitivity and specificity. The sensitivity and specificity of the prototype ELISA systems ranged from 77% to 99% and 89% to 100%, respectively [33–35]. Preliminary results on field plasma samples from cattle indicate a sufficient level of sensitivity (88.7%) and specificity (100%) for the indirect ELISA, and no cross-reactivity to *T. orientalis* was observed using the novel TaSP as an antigen. However, it should be noted that the relatively small number of plasma samples examined does not allow a definitive conclusion to be drawn on the diagnostic value of the novel recombinant antigen for the serological diagnosis of tropical theileriosis.

## Conclusion

Cloning the target nucleotide sequence into the pET19b vector using the ligase-free SLIM method followed by transformation and expression yielded a protein with a molecular weight of 32 kDa. Mass spectrometry analysis of the purified protein identified it as a surface protein of *T. annulata* (TaSP). Initial results using this recombinant TaSP as an antigen in the indirect ELISA were promising and support further evaluation of this test in larger numbers of cattle for its widespread use in the routine diagnosis of *T. annulata* infection and seroprevalence studies in cattle in Kazakhstan and possibly in neighboring countries.

## Data Availability

The supplementary data can be available from the corresponding author on a reasonable request.

## Authors’ Contributions

AR: Recombinant protein purification, ELISA protocol optimization, and PCR. AK: Obtained recombinant protein. NT: Carried out PCR, ELISA, and DNA isolation. MB: Sampling and DNA isolation. YM: Carried out PCR and ELISA. AS: Molecular investigations, data analysis, and drafted and revised the manuscript. CB: Data interpretation and critically reviewed, edited, and revised the manuscript. MK: Designed and supervised the study and drafted the manuscript. All authors have read, reviewed, and approved the final manuscript.
